# Blood miRNomes and transcriptomes reveal novel longevity mechanisms in the long-lived bat, *Myotis myotis*

**DOI:** 10.1186/s12864-016-3227-8

**Published:** 2016-11-10

**Authors:** Zixia Huang, David Jebb, Emma C. Teeling

**Affiliations:** UCD School of Biology and Environmental Science, University College Dublin, Belfield, Dublin, 4 Ireland

**Keywords:** Bats, Illumina miRNA-Seq, Blood miRNA, Comparative miRNome, *Myotis myotis*

## Abstract

**Background:**

Chiroptera, the bats, are the only order of mammals capable of true self-powered flight. Bats exhibit a number of other exceptional traits such as echolocation, viral tolerance and, perhaps most puzzlingly, extreme longevity given their body size. Little is known about the molecular mechanisms driving their extended longevity particularly at the levels of gene expression and post-transcriptional regulation. To elucidate the molecular mechanisms that may underlie their unusual longevity, we have deep sequenced 246.5 million small RNA reads from whole blood of the long-lived greater mouse-eared bats, *Myotis myotis*, and conducted a series of genome-wide comparative analyses between bat and non-bat mammals (human, pig and cow) in both blood miRNomes and transcriptomes, for the first time.

**Results:**

We identified 539 miRNA gene candidates from bats, of which 468 unique mature miRNA were obtained. More than half of these miRNA (65.1 %) were regarded as bat-specific, regulating genes involved in the immune, ageing and tumorigenesis pathways. We have also developed a stringent pipeline for genome-wide miRNome comparisons across species, and identified 37 orthologous miRNA groups shared with bat, human, pig and cow, 6 of which were differentially expressed. For bats, 3 out of 4 up-regulated miRNA (miR-101-3p, miR-16-5p, miR-143-3p) likely function as tumor suppressors against various kinds of cancers, while one down-regulated miRNA (miR-221-5p) acts as a tumorigenesis promoter in human breast and pancreatic cancers. Additionally, a genome-wide comparison of mRNA transcriptomes across species also revealed specific gene expression patterns in bats. 127 up-regulated genes were enriched mainly in mitotic cell cycle and DNA repair mechanisms, while 364 down-regulated genes were involved primarily in mitochondrial activity.

**Conclusions:**

Our comprehensive and integrative analyses revealed bat-specific and differentially expressed miRNA and mRNA that function in key longevity pathways, producing a distinct bat gene expression pattern. For the first time, we show that bats may possess unique regulatory mechanisms for resisting tumorigenesis, repairing cellular damage and preventing oxidative stresses, all of which likely contribute to the extraordinary lifespan of *Myotis myotis*.

**Electronic supplementary material:**

The online version of this article (doi:10.1186/s12864-016-3227-8) contains supplementary material, which is available to authorized users.

## Background

Of all mammals, bats possess some of the most unique and peculiar adaptations that render them as excellent models to investigate the mechanisms of extended longevity and potentially halted senescence. They are considered the ‘Methusalehs’^1^ among mammals due to their exceptional and surprising longevity given their body size and metabolic rate [1, 2]. Typically mammals that are small have a high metabolic rate (e.g. shrews) and do not live for a long time [1, 3]. However, despite their small size and high metabolic rate bats can live for an exceptionally long time, with the oldest recorded bat (wild caught as an adult) ever recaptured being >41 years old (*Myotis brandti*, 7 g, Brandt’s bat) [2, 4, 5]. Indeed, to get a positive correlation between longevity and body size in mammals, bats must be removed from the analyses [1]. By comparing the ratio of expected longevity to that predicted from the ‘non-bat placental mammal’ regression line (longevity quotient- LQ) only 19 species of mammals are longer lived than man, one of these species being the naked mole rat and the other 18 are bats [1]. This suggests that bats have some underlying mechanisms that may explain their exceptional longevity.

MicroRNA (miRNA) are a subset of short (~22 bp) endogenous non-coding RNA that play a significant role in post-transcriptional regulation, via repression of translation. Typically, the 5′ end of a mature miRNA (seed region) predominantly determines the specificity and sensitivity in binding the target mRNA through either perfect or partial complementarity to the mRNA 3′-UTR, resulting in mRNA degradation or translational repression [6]. Since the first miRNA, *lin-4*, was discovered in 1993 [7], a multitude of miRNA have subsequently been identified, and implicated in the regulation of the vast majority of biological pathways including cell cycle regulation, metabolism, tumorigenesis, as well as immune response. However, the role of miRNA regulation in mammalian ageing and the onset of age-related diseases has only recently been established [8].

In mammals, various miRNA have been shown to be differentially expressed during ageing, most of which appear to be generally tissue-specific [8]. It was reported that the expression of miR-93 remarkably increased in liver tissues from extremely old mice and rats [9, 10]. miR-93 targets glutathione-S-transferases (*GST*) and *SIRT1* genes [9, 10], which are vitally important in oxidative stress defense and metabolic homeostasis. Another example is miR-144. It is significantly up-regulated in aged human brains, which targets *ataxin-1*, the gene associated with spinocerebellar ataxin type 1 (SCA1) [11]. In addition to tissue-specific ageing, it is increasingly evident that many miRNA regulate gene expressions in well-known ageing pathways, most notably in the p53 tumor suppressor pathway (miR-34, miR-29 and miR-217, etc*.*) and insulin-like growth factor signaling pathway (IIS) (miR-17-92 family and miR-214, etc*.*) [8]. As altered patterns of miRNA expression can contribute to mammalian ageing, an investigation of the genome-wide miRNA profile (miRNome) may help elucidate the molecular mechanisms behind the extreme longevity of bats.

Despite being the second largest order of mammals (~1200 species) [12], there is a scarcity of genomic and transcriptomic bat resources. To date, only five well-annotated bat genomes (*Myotis lucifugus*, *Myotis brandtii*, *Myotis davidii*, *Pteropus alecto* and *Pteropus vampyrus*) are publically available. Phylogenomic studies of bat genomes and other mammalian species reveal that a number of genes are under positive selection in bats [5, 13, 14]. These genic adaptations have been correlated with traits such as echolocation, powered flight, hibernation, immunity and longevity. For example, specific non-synonymous mutations in *GHR* and *IGF1R*, key ageing-related genes, were detected in several long-lived vespertilionid bats (*M. brandtii*, *M. lucifugus* and *Eptesicus fuscus*) [5], while a large proportion of genes involved in DNA repair (*RAD50*, *KU80*, *MDM2* etc*.*) and the NF-кB pathway (*c-REL* and *ATM2* etc*.*) were reported to be under positive or divergent selection in *M. davidii* and *P. alecto* [14]. These results suggest bats may better detect and repair DNA damage. Intriguingly, positive selection was also detected in mitochondrial-encoded and nuclear-encoded oxidative phosphorylation genes in bats, which may explain their efficient energy metabolism necessary for flight [15]. Apart from comparative genome analysis, only a small number of transcriptomic studies on bats using mRNA-Seq and miRNA-Seq technologies have been carried out, focused primarily on the characteristics of hibernation [16], immunity [17, 18], echolocation [19] and phylogeny [20]. However, the molecular mechanisms of adaptations affecting longevity are still far from understood, especially with respect to gene regulation.

In the present study, we sequenced six small RNA libraries from whole blood sampled from wild-caught greater mouse-eared bats (*Myotis myotis*) and for the first time made genome-wide comparisons of both miRNomes and mRNA transcriptomes between bat and non-bat mammalian species (human, pig and cow). Whole blood is a nonlethal sample type that can reflect the real time overall physiological status of an individual [21]. Our earlier study showed that only a few drops of whole blood (<200 μl) contains a high percentage of genome-wide and body-wide transcripts [22]. We then compared both the corresponding bat blood miRNome and transcriptome to those of non-bat mammals (human, pig, cow), to elucidate bat-specific gene expression as well as post-transcriptional regulation.

The profiling of the *M. myotis* blood miRNome showed a large number of bat-specific miRNA involved in regulating important pathways related to immunity, tumorigenesis and ageing. Comparative analyses of both miRNomes and transcriptomes also revealed distinctive longevity mechanisms in bats. Several up-regulated miRNA possibly act as tumor suppressors. Gene Ontology (GO) enrichment analysis of differentially expressed protein-coding genes showed that up-regulated genes in bats compared to other mammals were mainly involved in mitotic cell cycle and DNA damage repair pathways while a high number of down-regulated genes were enriched in mitochondrial metabolism. The results and data presented here show unique regulatory mechanisms for protection against tumorigenesis, reduced oxidative stress, and robust DNA repair systems, likely contribute to the extraordinary longevity of bats.

## Results

### Bioinformatic analyses of *M. myotis* blood miRNome

**Fig. 1:**
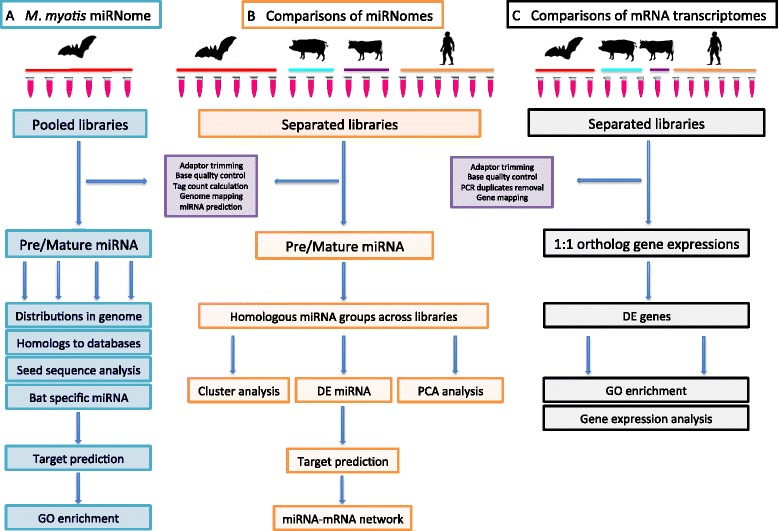
The workflow of analyses and bioinformatic pipelines. **a** The pipeline for identification and analyses of *M. myotis* blood miRNA. **b** The pipeline for comparisons and analyses of blood miRNomes between bat, human, pig and cow. **c** The pipeline for comparisons and analyses of blood mRNA transcriptomes between bat, human, pig and cow

We pooled the raw reads of all six libraries together (two individuals, three technical replicates each) to represent the *M. myotis* blood miRNome (Fig. 1a). A total of ~246.5 million single-end reads were generated on the Illumina HiSeq 2000 sequencer, with the uniform length of 50 bp. After adaptor trimming, size selection and base-calling filtering, we retained a final set of 202.9 million (82.3 %) high-quality post-processed reads for miRNA identification and further analysis. With strict criteria, the miRDeep2 pipelines predicted 539 pairs of mature miRNA and their corresponding precursors from which 203 were identified as known miRNA, with the remaining 336 predicted to be novel (Additional file 1: Table S1). As the same mature miRNA can be cleaved from different precursors, we acquired 468 unique mature miRNA after removing duplicates (Additional file 1: Table S2).

**Fig. 2:**
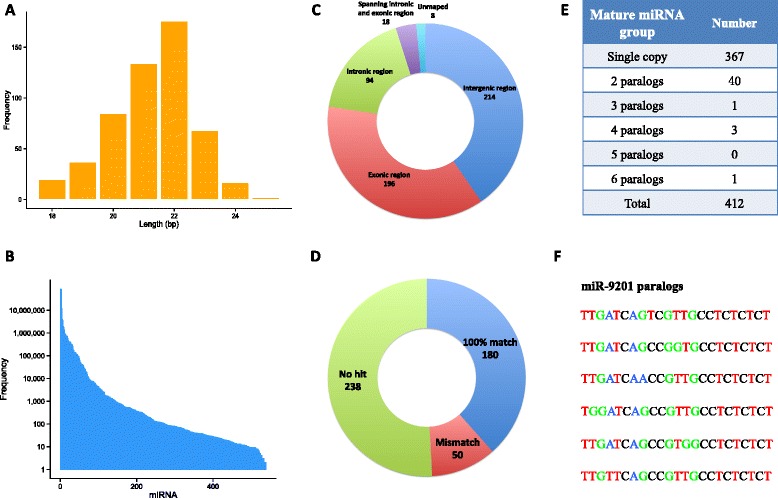
The bioinformatic analyses of the *M. myotis* blood miRNome. **a** The length distribution of *M. myotis* mature miRNA. **b** The frequency distribution of the *M. myotis* mature miRNA. **c** The genome locations of the miRNA genes (precursors). **d** The homology of the *M. myotis* mature miRNA to all the entries in miRBase (release 21). **e** The paralogous groups of *M. myotis* mature miRNA **f**) An example of the miRNA paralogous groups. The members in this paralogous group had consistent top BLAST hit, miR-9201

The bioinformatic analysis indicated that the miRNA (86.1 %) were mainly between 20 bp and 23 bp in length, with the peak at 22 bp (Fig. 2a), and their expression spanned several orders of magnitudes (Fig. 2b). The analysis of the genomic coordinates showed 214 miRNA (39.8 %) were located in the intergenic regions, followed by 196 (36.4 %) in the exonic regions as the second largest category (Fig. 2c). Interestingly, we also detected 18 miRNA traversing the boundaries of exons and introns. In order to annotate and evaluate the *M. myotis* blood miRNome, the predicted mature miRNA were compared to miRBase (release 21) and a collection of customized bat mature miRNA database (see Methods). Of all 468 unique mature miRNA, only 180 (38.5 %) and 166 (35.5 %) had 100 % identical entries in the miRBase and the customized bat miRNA database respectively, with more than half having no hits in both databases (Fig. 2d, Additional file 2: Figure S1). Typically, silencing of the target mRNA relies mainly on complementarity to bases 2–7 of mature miRNA, the ‘seed region’. We analyzed all seed regions of the blood miRNome, and the result revealed a set of 356 unique seeds, with the most frequent seed ‘gaggua’ and 68 other seeds appearing more than once. Not surprisingly, we found 29 novel seeds that did not exist in miRBase (release 21) (Additional file 1: Table S3).

**Fig. 3:**
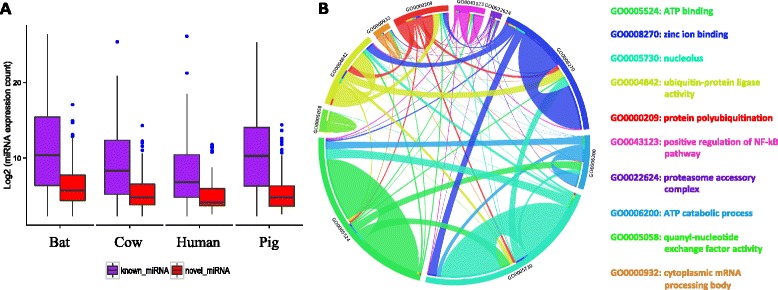
The analyses of bat-specific miRNA. **a** The distribution of the known miRNA and novel miRNA expression in bat, human, pig and cow, respectively. The expression data were *log*_*2*_ transformed. **b** The top 10 enriched GO terms of the targets of the bat-specific miRNA and their interaction networks. The networks show the number of targeted genes which are shared within different GO terms

We also ascertained the paralogous groups amongst the identified miRNA, resulting in 412 groups derived from 468 unique mature miRNA using our strict criteria (see Methods). 367 mature miRNA (78.4 %) were identified as single copy while 80 (17.1 %) were grouped into 40 pairs (Fig. 2e). We also found a group (miR-9201) containing 6 miRNA (Fig. 2f). To assess the validity of the procedure of paralog grouping, we validated all the members in 45 miRNA groups that had more than one member by BLAST-ing them against miRBase (release 21). Apart from 9 groups which had no hits, in 34 out of 36 groups (94.4 %) all members consistently had matching top BLAST hits in miRBase, suggesting our paralog grouping procedure was reliable in most cases. Furthermore, 351 miRNA genes (65.1 %) were discovered as bat-specific due to the failure to map to any of the 13 selected non-bat mammalian genomes (Additional file 1: Table S4, S5). However, these novel miRNA had a relatively lower level of expression than that of conserved miRNA, which appears to be a common phenomenon across species (Fig. 3a). The targets of these novel miRNA were subsequently predicted, and Gene Ontology enrichment analysis of the targets was carried out. Ranked by the FDR values, the most significantly enriched GO terms were ‘ATP binding’ (GO: 0005524), followed by ‘zinc ion binding’ (GO: 0008270) and ‘nucleolus’ (GO: 0005730). The top 10 enriched GO terms and their interaction networks were shown in Fig. 3b. However, it is a possibility that different species may use species-specific miRNA but target the same pathway. To ascertain the unique GO terms that bat-specific miRNA were involved in, we also analyzed the GO terms where the target genes of pig-, cow- and human-specific miRNA were enriched, respectively. By comparing the top 20 GO terms, we found that some common biological processes overlapped across species (bat, pig, cow and human), such as ‘ATP binding’ (GO: 0005524) and ‘zinc ion binding’ (GO: 0008270). However, we also noticed that many enriched GO terms were species-specific (Additional file 1: Table S6).

### Several up-regulated miRNA in bats may act as tumor suppressors

Interspecific gene expression variation of conserved miRNA can result from changes in selection pressures or through random genetic drift, which may explain the distinct patterns observed in bats. To identify and compare the conserved homologous miRNA across species, we employed the data from other studies focusing on the blood miRNome of human (*Homo sapien*), pig (*Sus scrofa*) and cow (*Bos taurus*) using miRNA-Seq technology. All the libraries (N_bat_ = 6, N_human_ = 6, N_pig_ = 3, N_cow_ = 3) were separately analyzed, and the same quality control steps and miRNA identification pipelines, which were used to identify the bat blood miRNome, were applied. The statistical information on each library is summarized in Additional file 1: Table S7.

**Fig. 4:**
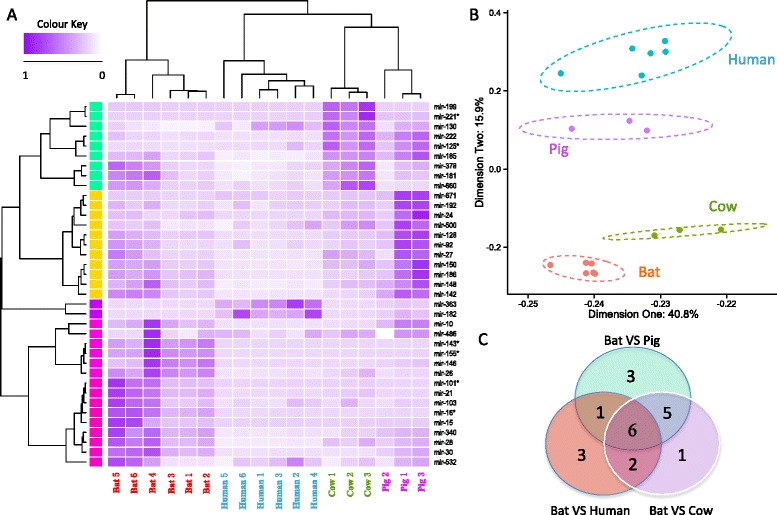
Comparative analyses of blood conserved miRNomes amongst bat, human, pig and cow. **a** Cluster analysis of conserved miRNA expression levels across all 18 libraries. The raw expression counts were normalized using the quantile method. The rows were clustered using Pearson correlation method (normal distribution, *P* > 0.05), while the columns were clustered using Spearman correlation method (non-normal distribution, *P* < 0.05). **b** Principal component analysis (PCA) of conserved miRNA expression levels across all 18 libraries. The raw expression counts were normalized using the quantile method. The proportion of the variance is indicated for each principal component. **c** The differentially expressed miRNA between bat and other mammalian species. 6 DE miRNA candidates were obtained after intersecting the results

**Fig. 5:**
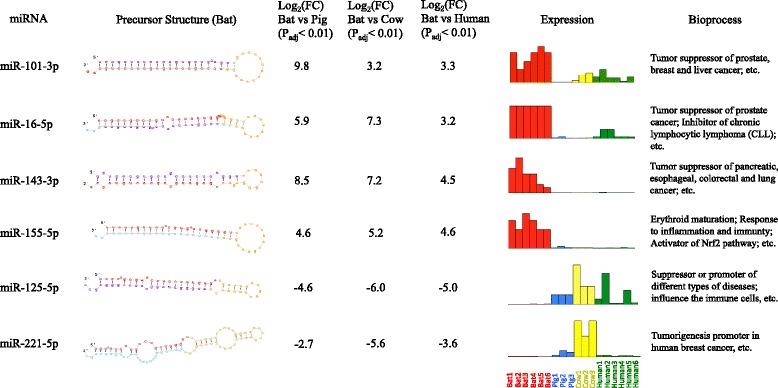
The summary of 6 differentially expressed (DE) miRNA. For the precursor structure, nucleotides highlighted in red indicate observed mature sequences, yellow for predicted stem loop region, blue for predicted star sequence and purple for observed star sequence. The fold changes between bat and other species were calculated by DESeq respectively. The relative miRNA expressions in each library were normalized using quantile normalization from the raw counts. The supporting references for the bioprocesses that miRNA are involved in are listed in the Additional file 2: Text S1

**Fig. 6:**
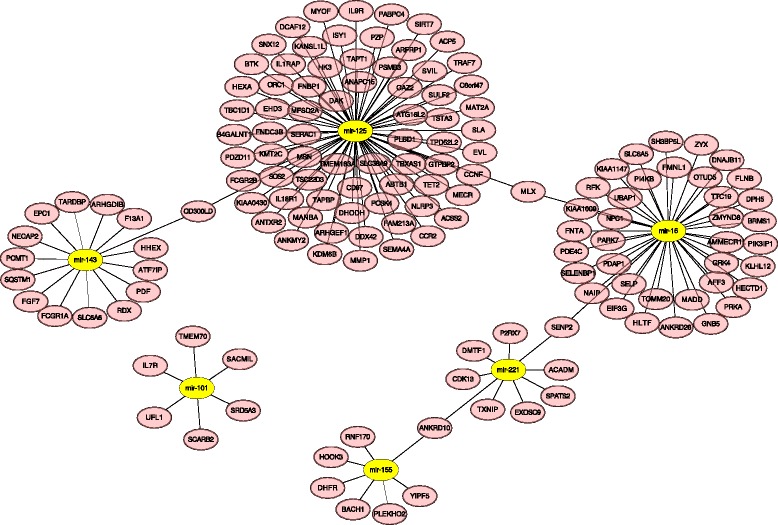
The interaction of 6 differentially expressed (DE) miRNA and their mRNA targets. The ellipses, highlighted in yellow, indicated the 6 DE miRNA, and the ellipses connected with the miRNA represent their mRNA targets

According to our stringent pipeline, only 37 conserved miRNA groups were identified across all libraries, 18 of which had more than one paralog in each library. To examine expression differences between species, we conducted a series of analyses on these 37 conserved miRNA groups, including hierarchical clustering analysis, principal component analysis (PCA) and differential expression (DE) analysis. The hierarchical clustering revealed several distinct miRNA expression patterns, suggesting their co-expression and common pathways they may be involved in (Fig. 4a). Not surprisingly, the libraries clustered together based on their respective species of origin (Fig. 4a). PCA also demonstrated that variations in the conserved miRNA expression differed primarily by species, exhibiting specific regions for each species on the plot (Fig. 4b). From the DE miRNA analysis, 12, 15 and 14 candidates were detected as significantly differentially expressed between bat and human, pig, cow respectively, with six DE miRNA common to all (Fig. 4c). The summary of the six DE miRNA common to all species is described in Fig. 5. Briefly, all DE candidates were single copy miRNA across all libraries, and 4 DE miRNA (miR-101-3p, miR-16-5p, miR-143-3p and miR-155-5p) were up-regulated in bats while 2 (miR-125-5p and miR-221-5p) were down-regulated. These DE miRNA are involved in a variety of biological pathways, but are primarily associated with cancer, immunity, apoptosis and ageing pathways. Given that a large abundance of protein-coding genes may be modulated by the same miRNA, we built a miRNA-mRNA interaction network using miRanda with strict filtering parameters (Fig. 6). In this network, 149 pairs of miRNA-mRNA were predicted, corresponding to 145 unique protein-coding genes regulated by 6 DE miRNA.

### Unique gene expression patterns indicate reduced respiratory activity and robust cellular maintenance systems in bats

**Fig. 7:**
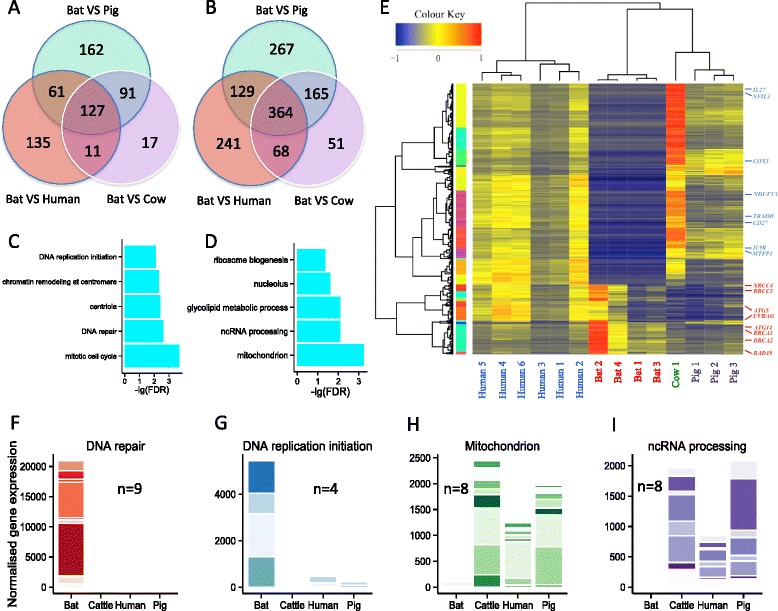
Comparative analyses of mRNA transcriptomes among bat, human, pig and cow. **a-b** The numbers of up-regulated and down-regulated genes in bat, respectively. The results of the three separate comparisons were intersected (bat *vs* human, bat *vs* pig, bat *vs* cow). **c-d** The top 5 Gene Ontology enriched terms of up-regulated and down-regulated genes in bat, respectively. The enriched terms with P_adj_-value < 0.05 were considered statistically significant. **e** The heatmap of 491 differentially expressed genes between bat and other mammals. The raw expression counts were normalized using the Trimmed of M-value (TMM) method. The rows were clustered using Pearson correlation method (normal distribution, *P* > 0.05), while the columns were clustered using Spearman correlation method (non-normal distribution, *P* < 0.05). **f**-**i** The relative accumulative gene expressions under certain enriched GO terms across bat, human, pig and cow. The raw expression counts were normalized using Trimmed mean of M-value (TMM) method, and the average gene expressions from the libraries of the same species were calculated and further summed based on the enriched GO terms

To better elucidate the molecular mechanisms underlying the many unique characteristics of bats, we also employed and analyzed the mRNA-Seq transcriptome data for these 4 species (N_bat_ = 4, N_human_ = 6, N_pig_ = 3, N_cow_ = 1). For bat, pig and cow, the blood mRNA transcriptomes were generated in the same studies corresponding to their blood miRNomes respectively, while human mRNA transcriptomes and miRNomes were employed from different sources (Table 1, Additional file 1: Table S8). In total, 3150 1:1 single-copy orthologous genes were extracted from their respective genomes, 3065 of which were retained after quality control (see Methods), and subsequently used for DE gene analysis. Analyzed by DESeq, 1366, 864 and 1166 genes were identified as DE genes between bat and human, pig, cow respectively (FDR < 0.05). Among those DE genes, 441, 216 and 364 genes were up-regulated while 802, 925 and 648 were down-regulated in bats compared to human, pig and cow (Fig. 7a and b). Overall, 127 genes were up-regulated and 364 genes were down-regulated in bats compared to other non-bat mammals (Fig. 7a and b). Gene Ontology enrichment analysis showed that up-regulated genes were mainly involved in DNA repair (GO: 0006281), DNA replication initiation (GO: 0006270) and mitotic cell cycle (GO: 0000278) while down-regulated genes were enriched in mitochondrion (GO: 0005739), glycolipid metabolic process (GO: 0006664) and ncRNA processing (GO: 0034470) (Fig. 7c and d).

**Table 1:** The publically available data employed for the comparative miRNomes and transcriptomes analyses in this study

	Comparative miRNomes			Comparative transcriptomes			
Species	Individual	Library	Data source	Species	Individual	Library	Data source
Bat (N_Ind_ = 2, N_Lib_ = 6)	B1	Bat1	this study	Bat (N_Ind_ = 2, N_Lib_ = 4)	B1		Huang, et al*.*
		Bat2^a^				Bat1^a^	
		Bat3^a^				Bat2^a^	
	B2	Bat4			B2		
		Bat5^a^				Bat3^a^	
		Bat6^a^				Bat4^a^	
Pig (N_Ind_ = 3, N_Lib_ = 3)	P1	Pig1^a^	Bao, et al*.*	Pig (N_Ind_ = 3, N_Lib_ = 3)	P1	Pig1^a^	Bao, et al*.*
	P2	Pig2^a^			P2	Pig2^a^	
	P3	Pig3^a^			P3	Pig3^a^	
Cow (N_Ind_ = 3, N_Lib_ = 3)	C1	Cow1	Bao, et al*.*	Cow (N_Ind_ = 3, N_Lib_ = 1)	C1	Cow1^b^	Bao, et al*.*
	C2	Cow2			C2		
	C3	Cow3			C3		
Human (N_Ind_ = 6, N_Lib_ = 6)	H1	Human1	Leidinger, et al*.*	Human (N_Ind_ = 6, N_Lib_ = 6)	H7	Human1	Memczak, et al*.*
	H2	Human2			H8	Human2	
	H3	Human3			H9	Human3	
	H4	Human4			H10	Human4	
	H5	Human5			H11	Human5	
	H6	Human6			H12	Human6	

^a^ in each line indicates miRNome and transcriptome libraries were generated from the same individual

^b^ indicates cow transcriptome was pooled from three individual libraries for sequencing

## Discussion

Mining miRNA from deep sequencing reads is highly dependent on the genome, which enables the prediction of their compact pre-miRNA hairpin structures. In this study, we employed the *M. lucifigus* genome as a proxy reference since the *M. myotis* genome has not yet been published. Although suffering some losses in the number, most abundant and conserved miRNA can be identified in a closely related species’ genome [23]. This is also shown by large number of miRNA genes (539) found in this study using the strict pipeline, the number of which falls within the range observed for other bat studies using miRNA-Seq, although different tissues and sequencing depths were used [17, 20]. The distribution of miRNA genes displayed no particular bias for genomic location. However, the largest category of miRNA (39.8 %) was in the intergenic regions (Fig. 2c), which is similar to human (49.9 %) and mouse (44.8 %) [24]. A large number of intragenic miRNA, which were located within introns and exons, were also found, and it is hypothesized that they may co-express with their host genes because of shared promoter sequences [25, 26]. Interestingly, we also detected 18 miRNA genes spanning the boundaries of exons and introns (Fig. 2c). These findings suggest the complexity of transcriptional splicing activity, and that the locations of miRNA can affect their expression and function. In addition, we identified and analyzed the paralogous groups in the *M. myotis* blood miRNome, observing that a large proportion of mature miRNA (78.4 %) were single-copy miRNA (Fig. 2e). Paralogous miRNA can shed light on gene duplication events, evolutionary history and post-transcriptional RNA editing. However, few studies on mammalian miRNA paralogs are available for comparison and analysis, probably due to the poor annotation of noncoding regions of mammalian genomes.

A significant proportion of miRNA (Fig. 2d) had no homologs in other species, reflected by many novel seed regions and bat-specific miRNA identified in this study. Potentially this could result from a lack of taxon-wide miRNA studies based on well-assembled and annotated genomes. However, a recent study estimated that the proportion of miRNA orthologous pairs between human and mouse was only 16 %, whose genomes are better annotated, leaving a large abundance of species-specific miRNA [24]. miRNA experience a rapid birth and death rate across species, and species-specific miRNA are considered to be evolutionarily recent. Not surprisingly, the expression level of these novel miRNA tended to be significantly lower (*P* < 0.05) than that of conserved miRNA (Fig. 3a). Abundantly expressed miRNA are highly likely to be functionally important, evolutionarily conserved, and under purifying selection; while novel miRNA with low expression level may have a comparatively weaker effect on their potential target transcripts, yet could convey species-specific adaptations. Occasionally, miRNA with low expression may be selectively favored and will be maintained in the genome. A direct correlation between the numbers of miRNA and biological complexity has been proposed, suggesting that miRNA innovation may play a key role in the emergence of increasingly novel and complex traits [27]. To understand the function of the bat-specific miRNA, we constructed the miRNA-mRNA network and performed function enrichment analysis on their targets. Many of the pathways and functions were associated with metabolism, immunity and protein homeostasis (Fig. 3b). Simultaneously, analyses of species-specific miRNA from other mammals (pig, cow and human) revealed common GO terms shared with bat, suggesting different, convergent species-specific miRNA can target the same pathways. The bat-specific GO terms, however, indicated the acquisition of novel miRNA in bats which regulate ageing and cellular senescence.

For bat, the most representative enriched terms, which are highly related to ageing and cell senescence, were ‘aging’ (GO: 0007568) and ‘guanyl-nucleatide exchange factor activity’ (GO: 0005085). Remarkably, some cell cycle mediators which accelerate cell growth and proliferation were included under the term ‘aging’, such as *GRB2*, *CASP7*, *SHIP1* and *GSN*. It is commonly accepted that accelerated cell growth directly coincides with tumorigenesis and cancer progression. Gelsolin (*GSN*), which functions as a cell growth promotor and apoptosis inhibitor, has been implicated in the oncogenesis of certain cancers [28]. Previous study showed its overexpression promoted the proliferation and mobility of hepatocellular carcinoma (HCC) cells [28]. Another example is *GRB2*, an activator of Ras/Raf/MEK/mitogen-activated protein kinase pathway (MAPK). It is reported that its downregulation could inhibit breast cancer cell growth [29]. Interestingly, we also noticed *SHIP1*, which limits blood cell production and immune regulatory cell numbers. The inhibition of *SHIP1* was shown to trigger apoptosis of blood cancer cells, suggesting the potential target for the treatment of hematological malignancies [30]. As well as inhibition of cell growth, bats may suppress inflammation and tumorigenesis by targeting guanyl-nucleotide exchange factors (GEFs). GEFs play a fundamental role in many diverse signaling pathways related to immunity, cell growth and tumorigenesis. RasGRP1, 2, 3 and SOS2 are all GEFs which activate Ras small GTPases and are some of the 50 GEFs targeted by bat-specific miRNA predicted in this study. RasGRP1 and 3 are recruited during the signaling cascades instigated by T-cell and B-cell receptor stimulation, respectively [31]. They are essential for the activation of Ras and promoting proliferation in T and B-cells in response to infection. Unsurprisingly, these genes have been shown to be overexpressed in a myriad of cancers, including melanoma, T-cell acute lymphoblastic leukemia (T-ALL) and prostate cancer [32, 33], making inhibitors of these proteins possible therapeutics. These results imply that *M. myotis* are capable of regulating cell cycle regulators as well as Ras small GTPases through degradation of direct or indirect activators. This may prevent tumor or cancer development and immune-related disorders, contributing to their long lifespan.

Comparisons of conserved miRNA expressions across species can also reveal distinct adaptations. However, there are two major obstacles to compare miRNA across species. First, there were limited miRNA resources that could be used for comparison. We employed data from human, pig and cow because they had both blood miRNomes and transcriptomes sequenced from healthy adults using NGST. This makes the investigation of the correlation between miRNA and mRNA expressions possible. Second, the existence of paralogs made it difficult to accurately quantify the orthologous miRNA across species [34]. Therefore, we calculated the geometric mean to represent the expression of each paralogous group. This is because all these paralogs were under strict inspection that they shared the same seed region, suggesting a high possibility of targeting the same mRNA. Due to a large number of bat-specific miRNA identified, it is not surprising that only 37 conserved orthologous groups were found across all 18 libraries. Cluster analysis and PCA indicated the differences within biological or technical replicates were much smaller than the differences between species (Fig. 4a, b).

Six conserved miRNA were found differentially expressed between bat and non-bat species (human, pig and cow), most of which were highly likely to be biologically functional in oncogenic or immune pathways. Among the 4 up-regulated miRNA, the expression of miR-101 in bat was at least three times than that in other mammals (Fig. 5). It is reported that miR-101, which functions as a tumor suppressor, is down-regulated in several cancers. Its overexpression inhibits cell proliferation and promotes apoptosis in breast cancer cells by inhibiting *Jak2* [35]. Similarly, expressions of miR-16 and miR-143 inhibit cell proliferation and suppress tumorigenesis, and miR-143 has been observed to be down-regulated in cervical cancer [36]. Importantly, one down-regulated miRNA, miR-221, was reported to function as a tumorigenesis promoter in human breast cancer. These findings demonstrate *M. myotis* are likely better at suppressing tumorigenesis than human, pig and cow, achieved by overexpression of miRNA associated with tumor suppression and inhibition of miRNA which promotes tumorigenesis. miR-155 is a multifunctional miRNA regulating inflammation, immunity and oncogenic pathways [37]. Interestingly, miR-155 also targets *Bach1*, a negative regulator of Nrf2, which is a core activator of the antioxidant response signaling pathway [38]. Activation of this pathway is a major mechanism for cells to defend against oxidative and electrophilic stresses by enhancing cellular antioxidant activity. This indicates that *M. myotis* efficiently degrade *Bach1* mRNA and activate the Nrf2 pathway, thus increasing defenses against cellular damage caused by reactive oxygen species (ROS). These miRNA regulatory mechanisms through which *M. myotis* can prevent tumorigenesis, inflammatory diseases and cellular oxidative damage, may explain their incredible longevity. Interestingly, transcriptome and proteome analysis of another long-lived mammal, the naked mole rat, also indicated their resistance to tumorigenesis, and insusceptibility to oxidative damage with age [39]. This demonstrates that long-lived mammals may regulate and control common ageing pathways to reach old age.

To address the uniqueness of bats from a different perspective, we also compared the RNA-Seq transcriptomes between bat and non-bat mammals. However, it is also a challenge to compare transcriptomes across species due to the existence of many paralogs. The quantification of paralogs calculated by mapping the clean reads to the genome cannot reflect the true expression, as paralogs are highly similar so that it is impossible to tease apart the reads having multiple mapping coordinates. Therefore, we only obtained all 1:1 single-copy orthologous protein-coding genes across bat, human, pig and cow genomes for analysis. Surprisingly, we identified many more down-regulated genes than up-regulated genes in bat (Fig. 7a, b). It was a concern that the quality of bat transcriptomes might be lower than that of other species, leading to the identification of massive down-regulated genes in bat. However, this concern was obviated by a measure of transcriptome completeness (CEGMA) which showed their qualities were highly comparable (bat: 64.9 %, human: 70 %, pig: 69 % and cow: 65.7 % in completeness).

Based on the enrichment results, a large number of mitochondria-related genes were down-regulated in bat (Fig. 7c, d). Mitochondria are the powerhouse of the cell generating adenosine triphosphate (ATP) from oxygen and the products of glycolysis. However, reactive oxygen species (ROS), such as superoxides and peroxides generated by the premature reduction of oxygen, can damage DNA, RNA and proteins, contributing to the pathophysiology of ageing. It is noteworthy that many of these down-regulated genes encode proteins, such as *NDUFV1* and *NDUFA1*, which are components of electron respiratory chain complex I, the primary site of ROS generation [40]. Reduced expression of mitochondrial-associated transcripts may represent a general mechanism where cells protect themselves against oxidative stress [41]. Our finding is consistent with the study of oxidative stress in another long-lived bat *M. lucifugus*, concluding that their mitochondria produce half to one-third the amount of hydrogen peroxide per unit of oxygen consumed than that of relatively short-lived mammals of similar size, short-tail shrew and white-footed mouse respectively [42].

On the other hand, *Myotis* bats seem to have evolved a heightened DNA repair system, showing up-regulation of several DNA repair genes. For example, *UVRAG*, encodes a protein which interacts with DNA repair enzymes to aid DNA double-strand break repair and plays a fundamental role in centrosomal and chromosomal stability [43]. Interestingly, UVRAG also interacts with Beclin 1 protein, leading to the activation of autophagy and inhibition of tumorigenesis [43]. The tumor suppressor genes *BRCA1* and *BRCA2*, which were both up-regulated in bats, encode proteins that are pivotal in maintaining genomic stability by promoting efficient and accurate repair of DNA double-strand breaks. Mutations in these two genes will lead to the development of various types of cancers [44]. We conclude that decreased levels of oxidative stress and heightened DNA repair activity may protect bats against genomic instability, contributing to halted ageing in the long-lived *M. myotis.*

## Conclusions

In summary, we established the first atlas of the blood miRNome of the long-lived bat, *M. myotis*, identifying a large number of miRNA involved in key ageing-related pathways. The comparative analyses of miRNomes and transcriptomes between bats and other mammals resulted in the discovery of many bat-specific miRNA, differentially expressed miRNA and mRNA, and their regulatory networks. Our results show that *M. myotis* exhibit increased protection against tumorigenesis and oxidative damage, heightened cellular damage repair activity and better genomic stability, all of which likely contribute to their exceptional longevity.

## Methods

### Bat blood sampling, total RNA extraction and miRNA sequencing

Two adult female greater mouse-eared bats (*Myotis myotis*) were captured in Béganne, western France (47°35′N, 2°14′W). For each bat, 80–200 μL of blood (depending on the weight of the individual) was collected from one of the two uropatagial veins. Total RNA was isolated from the whole blood using RNAzol**®** BD kit (catalog number RB192, Molecular Biology Center, Inc) according to the manufacturer’s instructions with minor modifications. Bat sampling, blood collection and total RNA extraction were carried out as described in our previous study [22]. The concentration and quality of total RNA were quantified and evaluated for each sample by a Nanodrop-ND-1000 Spectrophotometer (Thermo Scientific) and a 2100 Bioanalyzer (Aligent Technologies), respectively. RNA had a 280/260 ratio >1.8, RNA integrity number (RIN) > 8 and total RNA > 1 μg, and was further processed for Illumina deep sequencing. Six small RNA libraries from two 2-years-old adult female individuals were prepared and constructed from the total RNA using the TruSeq Small RNA Preparation Kit (Illumina) following the manufacturer’s protocols, and were sequenced to generate 50 bp single-end reads on HiSeq 2000 (Illumina, USA) platform by two different companies (Table 2). The raw small RNA reads have been deposited in National Center for Biotechnology Information (NCBI) under the Bioproject: PRJNA267654.

**Table 2:** Summary of bat miRNA sequencing and identification

Library	Bat1	Bat2	Bat3	Bat4	Bat5	Bat6	Pooled
Individual	B1	B1	B2	B2	B1	B2	
Sequencing Company	1	1	1	1	2	2	
No. of Raw Reads	24662730	44380583	28680178	93925331	27924234	26889119	246462175
No. of miRNA precursors	257	308	264	390	383	339	539
No. of unique mature miRNA	216	263	224	336	323	278	468

### Profiling of *M. myotis* miRNA

The workflow for miRNA identification and analyses is portrayed in Fig. 1a. The reads from the six libraries were pooled together to represent the blood miRNome of *M. myotis*. The identification of miRNA sequences was based mainly on the miRDeep2 pipeline. However, the raw reads were initially inspected and filtered as a quality control step. Briefly, the 3′ adaptor sequence (TGGAATTCTCGGGTGCCAAGGAACTCCAA) was cut using cutadapt (v1.3) [45], and only the trimmed reads with the read lengths between 18 and 25 bp were preserved, and further filtered by base quality using NGS-toolkit (v2.3) [46]. The sequences containing unknown bases (N’s) were discarded, and those with at least 80 % of bases, with quality scores above Q30, were retained as clean reads. The identical clean reads were compressed to single entries with the headers designating their frequencies using Mapper.pl, a module of miRDeep2 pipeline [47]. The unique sequence tags were then mapped to the genome and analyzed by miRDeep2.pl to predict mature miRNA and their corresponding precursors. As the genome of *M. myotis* is not available, the well-annotated *M. lucifugus* genome was employed in this study. The predicted miRNA candidates were filtered by the tag count and the probability of true positive estimated by miRDeep2. The candidates, with a count less than 5 and true positive probability lower than 60 %, were deemed unreliable and were excluded from the further analysis.

### Bioinformatic analyses of *M. myotis* miRNome

The locations of predicted miRNA genes were determined by mapping the precursors to exonic, intronic and intergenic regions of the *M. lucifugus* genome using bowtie (v 0.12.7) [48], allowing no mismatches (bowtie -v 0 --best). The exonic, intronic and intergenic regions were extracted from the genome based on the annotation information using Bedtools (v.2.4.3) [49]. In order to better annotate the miRNome, we collected bat miRNA from multiple studies, including *P. alecto* [17], *M. lucifugus* [20] and *E. fuscus* [20]. Mature miRNA were respectively compared to the miRBase (release 21) [50] and this customized bat miRNA dataset using BLASTN, with the parameters optimized for short read alignment (−evalue 0.1 -strand plus -word_size 4 -gapopen −8 -gapextend −6 -penalty −4) [51]. Based on their top BLAST hits, miRNA were categorized into the entire match, mismatch and no-hit groups. The entire match is defined by allowing maximum 2 bp overhanging at both ends for each sequence, while the mismatch group only allows 1 bp overhanging at each end but can tolerate 1 bp mismatch or indel. The sequences with other conditions of alignments and no hits were categorized into the no-hit group. The seed sequences of predicted *M. myotis* miRNA were analyzed and novel seeds discovered by comparing all seed sequences to those of all mature miRNA in the miRBase database (release 21). To identify groups of paralogous mature miRNA in the *M. myotis* miRNome, we performed sequence clustering analysis using CD-HIT [52] based on the sequence similarity. The parameters were set as ‘-c 0.9 -aL 0.9’, signifying that the sequences would cluster into groups if they share at least 90 % of both similarities and sequence overlaps in length. These preliminary paralogous groups were manually inspected, and the final set of paralogous groups was determined by excluding the alignments with mismatches > 2 and overhanging > 1 at each end. Each group was validated by comparing its members to the miRBase database (release 21) using miRBase BLAST web service, and their top BLAST hits were inspected.

In addition, bat-specific miRNA genes were determined by mapping all predicted miRNA precursors to 13 non-bat mammalian genomes (Additional file 1: Table S4) using bowtie (v0.12.7), allowing 3 mismatches per alignment. These 13 non-bat mammals were selected to represent the vast ecological and evolutionary diversity within mammals, and their genomes were also sequenced, assembled and annotated to a high standard. miRNA genes that did not map to any of the genomes above were considered to be bat-specific. The targets of these bat-specific miRNA were also predicted and analyzed. Briefly, we obtained the assembled *M. myotis* blood mRNA-Seq transcriptome from our previous study [22], which also had corresponding miRNome sequenced by Illumina for each individual used in this study (Table 1). The 3′-UTR sequences were extracted from the assembled transcripts which had been annotated against all protein-coding genes in the *M. lucifugus* genome and further filtered to discard those with lengths shorter than 20 bp. The miRNA-mRNA interactions were then predicted using miRanda [53]. Gene Ontology enrichment analysis of target genes was performed using Blast2GO suite [54], with the *M. lucifugus* genome as the background reference. The enriched GO terms with false discovery rates (FDR) < 0.05 were considered statistically significant. The interaction networks of GO terms were generated using Circos web service [55]. The human-, pig- and cow-specific miRNA were also respectively identified using the same pipeline described above.

### Comparative miRNomes between bat, human, pig and cow

In order to ascertain the potential species-specific adaptations in long-lived bats, genome-wide miRNA expressions in *M. myotis* blood were analyzed and compared to other mammals. In this study, six human (*H. sapien*), three cow (*B. taurus*) and three pig (*S. scrofa*) blood miRNA-Seq libraries [56, 57] were employed as non-bat mammal comparisons. All individuals used were healthy adults when the blood was collected. The raw small RNA data from human, pig and cow were downloaded from NCBI SRA database (Additional file 1: Table S7), and the pipeline for comparative miRNome analyses is displayed in Fig. 1b. For each library (N_bat_ = 6, N_human_ = 6, N_pig_ = 3, N_cow_ = 3), miRNA were processed and predicted through the same quality control and identification pipelines described previously. To obtain the differentially expressed (DE) miRNA, the conserved miRNA were required to be identified amongst bat, human, pig and cow. To do this, the mature miRNA sequences from 18 libraries were merged and subsequently clustered using CD-HIT with the same parameters as above. The homologous miRNA clusters were retrieved and filtered using the same criteria as the identification of paralogous groups but not allowing any mismatches within the seed region. Only homologous miRNA, which were discovered across all 18 libraries, were considered to be conserved miRNA and associated expression data (tag counts) were collected. However, there were some conserved miRNA which had paralogs in each library, leading to inaccurate quantifications of miRNA. Under this circumstance, we calculated the geometric mean of the expression from these paralogs to represent the expression of this conserved group across each library. The formula of geometric mean is described below, where ***x̄***_***g***_ represents the geometric mean; ***x***_***n***_ represents the expression of each member in a certain paralogous group; ***k*** is the number of the members in that group.

$$ {\overline{x}}_g={\left({\displaystyle \prod_{n=1}^k{x}_n}\right)}^{\frac{1}{k}} $$

After obtaining the matrix of conserved miRNA, and their associated expression data in each library, tag counts were normalized using quantile normalization before further analysis. Hierarchical cluster analysis and principal component analysis (PCA) were then implemented using the R packages *hclust* and *prcomp* respectively. For cluster analysis, the miRNA (rows) were clustered using Pearson correlation method (normal distribution of the data; *P*-value > 0.05) while the libraries (columns) were clustered using Spearman correlation method (non-normal distribution of the data; *P*-value < 0.05). DE miRNA were identified between bat/human, bat/pig and bat/cow using the DESeq’s generalized linear model (GLM) [58]. DE miRNA required an absolute value of log_2_ (Fold Change) > 2 and a Benjamin-Hochberg adjusted *P*-value < 0.01, denoting a 99 % probability that each miRNA was differentially expressed to be deemed statistically significant. The overlapping DE miRNA from bat/human, bat/pig and bat/cow were collected, and further investigated. Their mRNA targets were predicted using miRanda.

### Comparative mRNA transcriptomes between bat, human, pig and cow

To better understand the expression difference between bats and other mammals, we also employed and analyzed the mRNA-Seq data of the blood transcriptome from these four species (N_bat_ = 4, N_human_ = 6, N_pig_ = 3, N_cow_ = 1; libraries from cow were pooled prior to sequencing; Table 1) [22, 56, 59]. The raw mRNA-Seq data of bat, human, pig and cow were obtained from NCBI SRA database (Additional file 1: Table S8), and the workflow for comparative transcriptome analyses is described in Fig. 1c. For the quality control steps, the raw reads were trimmed of adaptor sequences, filtered by base quality and PCR duplicates were removed. Subsequently, the clean reads were pooled by species, assembled, and annotated by their respective genomes. See Additional file 1: Table S4 for genome build details. In this study, the *M. lucifugus* genome (v2.0) was employed as a reference since the *M. myotis* genome is not available, and the pipelines applied were as described in our previous study [22]. The quality of assembled transcripts for each species was assessed by the Core Eukaryotic Gene Mapping Approach (CEGMA) system [60].

For the analysis of DE genes, 1:1 single-copy orthologous genes in the genomes of *M. lucifugus*, *H. sapiens*, *S. scrofa* and *B. taurus* were retrieved using the ensembl web-tool BioMart [61]. For each library (N_bat_ = 4, N_human_ = 6, N_pig_ = 3, N_cow_ = 1), these orthologous genes were quantified using Sailfish (v0.7.6) with the default parameters [62]. The raw count of each gene in each library was collected and the genes that were not expressed in any libraries were excluded from DE gene analysis. The raw counts were normalized using Trimmed Mean of M-value (TMM) method [63], and DE genes were detected between bat and other mammals respectively, using the DESeq’s generalized linear model (GLM). The genes, which had an absolute value of log_2_ (Fold Change) > 2 and a Benjamin-Hochberg adjusted *P*-value < 0.05, were considered as significant DE genes. The intersections of up-regulated and down-regulated genes between bat and other mammals were analyzed. The Gene Ontology enrichment analysis was conducted using Blast2GO suite. Due to the large number of enriched GO terms obtained, an additional filter of Benjamin-Hochberg adjust *P*-value < 0.05 was applied.

## Additional files

Additional file 1: Contains supplementary **Table S1-S11. Table S1.** The miRNA precursor sequences and their corresponding mature miRNA sequences predicted from *M. myotis* blood. **Table S2.** The unique mature miRNA sequences. **Table S3.** The novel seed regions identified from the mature miRNA sequences. **Table S4.** The genomes of selected species used for the identification of bat-specific miRNA, and their mapping results. **Table S5.** The list of predicted bat-specific miRNA **Table S6.** The unique enriched GO terms of the targets of the species-specific miRNA (bat, human, pig and cow). **Table S7.** The summary of the miRNA-Seq data used in this study. **Table S8.** The summary of the mRNA-Seq data used in this study. **Table S9.** The list of the predicted species-specific miRNA sequences (human, pig and cow). **Table S10.** The orthologous miRNA expression data (raw counts) across bat, human, pig and cow. **Table S11.** The single-copy orthologous gene expression data (raw counts) across bat, human, pig and cow. (XLSX 370 kb)
Additional file 2: Contains supplementary **Figure S1** and **Text S1. Figure S1.** The number of homology of *M. myotis* mature miRNA to the customized bat miRNA dataset. **Text S1.** The supporting references for the bioprocesses that 6 differentially expressed (DE) miRNA are involved in. (PDF 176 kb)

## Abbreviations

CEGMA: Core eukaryotic genes mapping approach
DE: Differentially expressed
FDR: False discovery rate
GEF: Guanyl-nucleotide exchange factor
GLM: Generalized linear model
GO: Gene ontology
GST: Glutathione-S-transferase
HCC: Hepatocellular carcinoma
IIS: Insulin-like growth factor signaling pathway
MAPK: Ras/Raf/MEK/mitogen-activated protein kinase pathway
miRNA: microRNA
NCBI: National center for biotechnology information
NF-кB: Nuclear factor kappaB signaling pathway
NGST: Next generation sequencing technology
PCA: Principal component analysis
ROS: Reactive oxygen species
SRA: Sequence read archive
T-ALL: T-cell acute lymphoblastic leukemia
TMM: Trimmed mean of M-value
UTR: Untranslated region

## Footnotes

1 Methusaleh was a biblical character said to have lived for 969 years.
