# Supplementary material for: Blood miRNomes and transcriptomes reveal novel longevity mechanisms in the long-lived bat, Myotis myotis
Source: BMC Genomics. 2016 Nov 10;17:906. doi: 10.1186/s12864-016-3227-8 (PMC5103334; doi:10.1186/s12864-016-3227-8)
Supplement: Additional file 2: — Contains supplementary Figure S1 and Text S1. Figure S1. The number of homology of M. myotis mature miRNA to the customized bat miRNA dataset. Text S1. The supporting references for the bioprocesses that 6 differentially expressed (DE) miRNA are involved in. (PDF 176 kb) [file 12864_2016_3227_MOESM2_ESM.pdf]

Figure S1. The number of homology of *M. myotis* mature miRNA to the customized bat miRNA database.

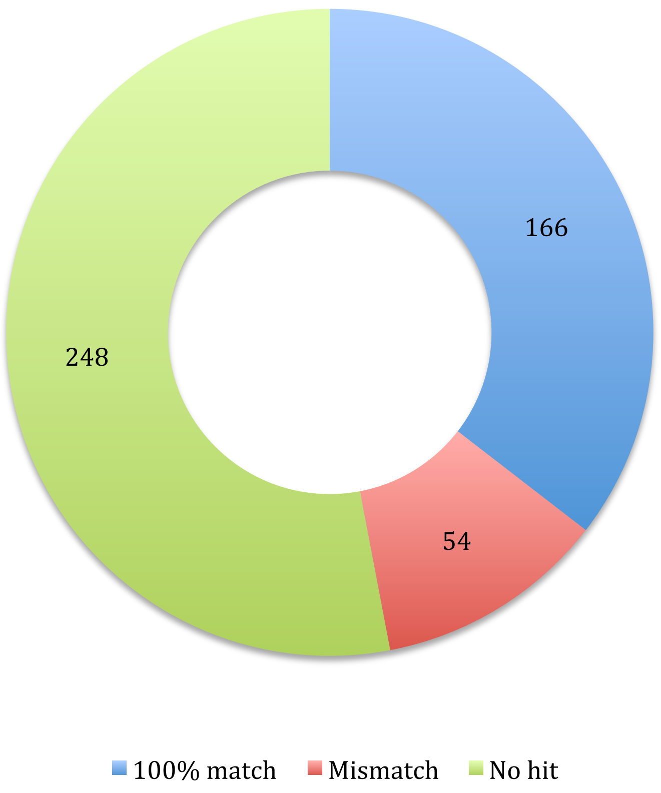

Text S1. The supporting references for the bioprocesses that 6 DE miRNA are involved in.

miR-101-3p [1-3]

miR-16-5p [4, 5]

miR-143-3p [6-9]

miR-155-5p [10, 11]

miR-125-5p [12, 13]

miR-221-5p [14, 15]

1. Shen Q, Bae HJ, Eun JW, Kim HS, Park SJ, Shin WC, Lee EK, Park S, Park WS, Lee JY: **MiR-101 functions as a tumor suppressor by directly targeting nemo-like kinase in liver cancer.** *Cancer letters* 2014, **344:**204-211.
2. Hao Y, Gu X, Zhao Y, Greene S, Sha W, Smoot DT, Califano J, Wu TC, Pang X: **Enforced expression of miR-101 inhibits prostate cancer cell growth by modulating the COX-2 pathway in vivo.** *Cancer Prevention Research* 2011, **4:**1073-1083.
3. Wang L, Li L, Guo R, Li X, Lu Y, Guan X, Gitau SC, Xu C, Yang B, Shan H: **miR-101 promotes breast cancer cell apoptosis by targeting Janus kinase 2.** *Cellular Physiology and Biochemistry* 2014, **34:**413-422.
4. Bonci D, Coppola V, Musumeci M, Addario A, Giuffrida R, Memeo L, D'Urso L, Pagliuca A, Biffoni M, Labbaye C: **The miR-15a–miR-16-1 cluster controls prostate cancer by targeting multiple oncogenic activities.** *Nature medicine* 2008, **14:**1271-1277.
5. Pekarsky Y, Croce CM: **Role of miR-15/16 in CLL.** *Cell Death & Differentiation* 2015, **22:**6-11.
6. Hu Y, Ou Y, Wu K, Chen Y, Sun W: **miR-143 inhibits the metastasis of pancreatic cancer and an associated signaling pathway.** *Tumor Biology* 2012, **33:**1863-1870.
7. Ansari MH, Irani S, Edalat H, Amin R, Roushandeh AM: **Deregulation of miR-93 and miR-143 in human esophageal cancer.** *Tumor Biology* 2015:1-7.
8. Kent OA, McCall MN, Cornish TC, Halushka MK: **Lessons from miR-143/145: the importance of cell-type localization of miRNAs.** *Nucleic acids research* 2014, **42:**7528-7538.
9. Zhang N, Su Y, Xu L: **Targeting PKCε by miR-143 regulates cell**

apoptosis in lung cancer. *FEBS letters* 2013, **587:**3661-3667.

10. Faraoni I, Antonetti FR, Cardone J, Bonmassar E: **miR-155 gene: a typical multifunctional microRNA.** *Biochimica et Biophysica Acta (BBA)-Molecular Basis of Disease* 2009, **1792:**497-505.
11. Li S, Chen T, Zhong Z, Wang Y, Li Y, Zhao X: **microRNA-155 silencing inhibits proliferation and migration and induces apoptosis by upregulating BACH1 in renal cancer cells.** *Molecular medicine reports* 2012, **5:**949-954.
12. Sun Y-M, Lin K-Y, Chen Y-Q: **Diverse functions of miR-125 family in different cell contexts.** *J Hematol Oncol* 2013, **6.**
13. Pan W, Zhu S, Dai D, Liu Z, Li D, Li B, Gagliani N, Zheng Y, Tang Y, Weirauch MT: **MiR-125a targets effector programs to stabilize Treg-mediated immune homeostasis.** *Nature communications* 2015, **6.**
14. Garofalo M, Quintavalle C, Romano G, Croce CM, Condorelli G: **miR221/222 in cancer: their role in tumor progression and response to therapy.** *Curr Mol Med* 2012, **12:**27-33.
15. Shah MY, Calin GA: **MicroRNAs miR-221 and miR-222: a new level of regulation in aggressive breast cancer.** *Genome Med* 2011, **3:**56.
